# Breaking Vendors and City Locks through a Semantic-enabled Global Interoperable Internet-of-Things System: A Smart Parking Case

**DOI:** 10.3390/s19020229

**Published:** 2019-01-09

**Authors:** Pablo Sotres, Jorge Lanza, Luis Sánchez, Juan Ramón Santana, Carmen López, Luis Muñoz

**Affiliations:** Network Planning and Mobile Communications Lab, Universidad de Cantabria, Edificio de Ingeniería de Telecomunicaciones, Plaza de la Ciencia s/n, 39005 Santander, Spain; psotres@tlmat.unican.es (P.S.); jlanza@tlmat.unican.es (J.L.); jrsantana@tlmat.unican.es (J.R.S.); clopez@tlmat.unican.es (C.L.); luis@tlmat.unican.es (L.M.)

**Keywords:** interoperability, Internet of Things, semantics, Smart City, pilot project

## Abstract

The Internet of Things (IoT) is unanimously identified as one of the main technology enablers for the development of future intelligent environments. However, the current IoT landscape is suffering from large fragmentation with many platforms and vendors competing with their own solution. This fragmented scenario is now jeopardizing the uptake of the IoT, as investments are not carried out partly because of the fear of being captured in lock-in situations. To overcome these fears, interoperability solutions are being put forward in order to guarantee that the deployed IoT infrastructure, independently of its manufacturer and/or platform, can exchange information, data and knowledge in a meaningful way. This paper presents a Global IoT Services (GIoTS) use case demonstrating how semantic interoperability among five different smart city IoT deployments can be leveraged to develop a smart urban mobility service. The application that has been developed seamlessly consumes data from them for providing parking guidance and mobility suggestions at the five locations (Santander and Barcelona in Spain and Busan, Seoul and Seongnam in South Korea) where the abovementioned IoT deployments are installed. The paper is also presenting the key aspects of the system enabling the interoperability among the three underlying heterogeneous IoT platforms.

## 1. Introduction

Smart cities have the potential to be a main motivator in developing a global Internet of Things (IoT) market of services and hardware. Nonetheless, the emergent smart city scenario is encountering specific challenges that are hindering its growth, obstructing fast innovation and preventing widespread market adoption. One of these key challenges is the implementation of solutions that enable scaling city platforms without vendor and/or city lock-in.

On the one hand, cities find it difficult to invest into a specific solution given the lack of well-established standards and an interoperable marketplace for IoT-enabled smart city solutions as it would create dependencies on a single provider. There is a fear of vendor lock-in that could jeopardize future IoT deployments enlarging the underlying infrastructure if they imply significant system integration expenses. Consequently, city managers are often reluctant to make larger investments in smart infrastructure for cities, thus, hampering the market uptake. On the other hand, current APIs for accessing the data that streams from IoT infrastructures show a large heterogeneity across the platforms that different cities are using, and so can vary the availability of information sources and data formats. Developers and providers of IoT-based smart city services face many challenges to deploy and run a service, which has been implemented for one city, to another, thus reducing the benefits that come from economies of scale. We call this fear of “city lock-in”.

The IoT full potential is built around the concept of smart devices that can discover their context and build collaborations with other smart devices and services around them to create value. Discovery, understanding and collaboration involves something more than just the ability to communicate and to exchange data. Interoperability is typically associated to “the ability of two or more systems or components to exchange data and use information” [1]. However, what is necessary to bring the IoT vision to a truly interoperable scenario involves also the so-called semantic interoperability, which “means enabling different agents, services, and applications to exchange information, data and knowledge in a meaningful way” [2].

In this respect, standardization fora like OneM2M (http://www.onem2m.org), international communities like FIWARE (https://www.fiware.org) and research projects like BIG-IoT (http://big-iot.eu/) are defining open standards and ecosystems that establish a common playground for global and interoperable solutions to flourish. However, even after these homogenization initiatives there is still a risk of fragmentation that might cause that the combination of different data streams into one single service becomes a serious challenge for developers. On the one hand, an easy and efficient way to discover and retrieve all this information is needed. On the other hand, translations between the different information models available on each platform are not trivial.

In this paper, we present a smart parking use case that is built around a mobile application that we have implemented for consuming data from five different and heterogeneous smart city deployments located in the cities of Santander and Barcelona (both in Spain), and Busan, Seoul and Seongnam (all three in South Korea). The main objective of the field trial presented in this paper is to serve as a validator of Global IoT Services (GIoTS), enabling transparent user roaming between the five cities involved in the pilot. The use case implementation and its trialing over real-world smart city deployments are the way for validating the second key contribution from this paper. This is, in fact, the actual technical novelty that is included in our work. Integrating a system for enabling interoperability between oneM2M FIWARE and BIG-IoT platforms is the second result that is presented in this paper. In this sense, the specification and implementation of an NGSIv2 GW Service for the BIG-IoT platform is the actual enabler for this integration.

The definition and integration of a system enabling seamless consumption of context information gathered by heterogeneous IoT platforms represents a major technical advance for preventing vendor lock-in as well as city lock-in. Regarding the vendor lock-in, we have been able to integrate both standard-based platforms (i.e., oneM2M and FIWARE-based ones) and proprietary ones (i.e., Barcelona’s one). This demonstrates that infrastructure from different vendors can be used for the same use-case, thus, counteracting the vendors’ lock. Concerning the city lock-in, we have demonstrated that the same application can be used across cities, thus benefiting from economies of scale and enlarging potential impact of investments made on smart city services. Last but not least, technical novelty of our solution lays also on the use of semantics to enable the required interoperability among platforms.

It is important to mention that this paper is an extended version of [3]. The key additions included in this paper are the discussion of key related work in the area of IoT interoperability, the addition of three additional cities to the use case, the integration of the BIG-IoT platform [4] to the system and the extension of the mobile application to serve the users requirements and consume the newly available information. The elicitation of the use-case requirements and the binding between them and the components leveraged from the Wise-IoT (http://wise-iot.eu/en/home/) and BIG-IoT projects is the last of our contributions. In this respect, some of the requirements that have been fulfilled for supporting the use case presented in this paper were not addressed in our previous work. These newly supported requirements mainly relate to the discoverability of the context information. They have been covered thanks to our work integrating the BIG-IoT solution.

The remainder of the paper is organized as follows: Section 2 presents a modelling and review of the IoT interoperability solutions that have been proposed to address the IoT landscape lack of coherence challenge. Moreover, it reviews the key features from the Wise-IoT and BIG-IoT projects with focus on the tools and solutions that we have used for the system that we have integrated in order to support the use case that is described in the paper. In Section 3, a short introduction to the physical deployments in the five cities where the use case takes place is given. Once the characteristics of the physical deployments have been introduced, in Section 4, the key use case requirements are introduced and bound to the main features of the Wise-IoT and BIG-IoT platforms. The description of the system that has been integrated to support the smart parking use case, together with the brief sketching of the platforms and components that are part of it is presented in Section 5. Section 6 describes the key features of the mobile application that has been implemented to support the smart parking use case. Finally, Section 7 concludes the paper.

## 2. Related Work

### 2.1. Interoperability Solutions

Heterogeneity at any level (device, networking, middleware, application service, data and semantics) is one of the critical characteristics of current IoT scenarios. In fact, it is preventing IoT solutions from easily interoperating [5]. For these systems to enable building new innovative applications, which make usage of data from different existing vertical IoT silos, they have to not only exchange information but also have a common understanding of the significance of this data. This implies, regardless of whether the present IoT frameworks will uncover their information and assets to others, that orthogonal data models become a big problem when they have to interoperate as they have differing definitions or even meanings of the reality reported by the IoT infrastructure.

**Figure 1:**
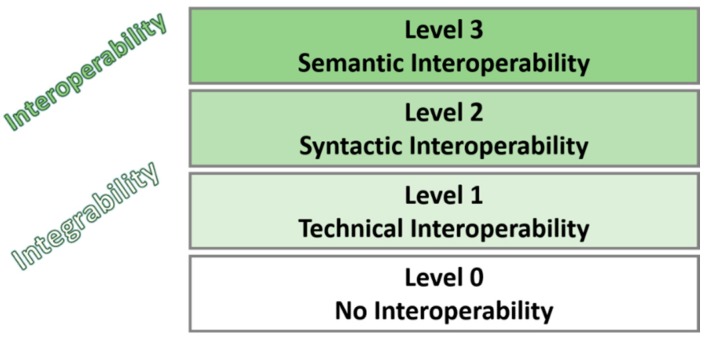
Lower levels of Conceptual Interoperability Model (from [7]).

“Broadly speaking, interoperability can be defined as a measure of the degree to which diverse systems, organizations, and/or individuals are able to work together to achieve a common goal” [6]. In order to narrow down this definition, we will refer to the Levels of Conceptual Interoperability Model (LCIM) [7] depicted in Figure 1. LCIM was created for a completely different scope but it can be applied for structuring the different approaches taken for the IoT interoperability. In this sense, we will focus on the three first levels of LCIM where Level 1 relates to the low-level technical connectivity of platforms, Level 2 to employing shared languages or protocols like JSON or MQTT and Level 3 to having a common understanding of the information that is being shared. The three upper levels are not addressed as they are mainly related to the original scope of LCIM (i.e., simulation theory). They are focused on how the data is used, how this data change the state of the systems consuming it and how systems can be composed respectively.

#### 2.1.1. Technical Interoperability Solutions

As the IoT is a developing technology with no already established coordination, we are witnessing the creation and proposition of many solutions and (pseudo) standards. Moreover, it is foreseen that this situation will continue in the coming years leading to even higher heterogeneity. In reality, a wide range of (semi) norms do currently exist in the IoT field from covering different perspectives and, as a result, addressing sectoral issues that allude to universes that do not talk between themselves.

Several communication solutions are currently used at the device level. Well-established wireless networks (i.e., 4G and Wi-Fi) are progressing to address the IoT specific requirements. Moreover, there are specially tailored protocols and mechanisms for sensors and actuators (e.g., IEEE802.11ah or IEEE 802.15.4) together with other proprietary Low Power Wide Area Network (LPWAN) solutions (e.g., SIGFOX, LoRaWAN). At the network level, 6LoWPAN [8] is probably the most well-known example of a protocol aimed at unifying the interconnectivity of sensor networks to the Internet. However, there are a number of solutions both for encapsulation (e.g., 6TiSCH [9], 6Lo [10]) and for routing (e.g., RPL [11], CORPL [12]). Moreover, at the middleware level, there is a similar scenario with a large number of competing solutions [13] including the “big players” in cloud computing-based infrastructures (e.g., Amazon AWS IoT (https://aws.amazon.com/es/iot/), Google Cloud IoT (https://cloud.google.com/solutions/iot/), Xively (https://xively.com/), Azure IoT (https://azure.microsoft.com/ en-us/overview/iot/)).

This heterogeneous and complex landscape is jeopardizing large-scale diffusion that needs clear use-cases and ease of use solutions [14]. The first level of homogenization solutions is typically proposed through the utilization of gateways [15,16]. In this sense, literature is full of proposals [17] leveraging the ubiquitous presence of smartphones and the fact that they already have multiple radio interfaces. These solutions [18,19] stands on the idea that thanks to these features, smartphones can perform as gateways to gather, process and forward data generated by IoT devices.

The main problem with all these solutions, independently of the level at which they are lying is that they can only address the basic functionality of collecting the data but they still do not enable seamless service creation out of this data. The service provider will still have to deal with, for example, temperature information that comes in different format (e.g., binary, XML or JSON), with different meaning (e.g., Celsius, Fahrenheit or Kelvin) and related to different domain (e.g., weather or health). With this in mind, it will be tricky for this provider to generate a weather report after getting data from Bluetooth body-worn thermometer, LoRa weather station and Wi-Fi engine temperature probe. Technical interoperability solutions can make all these pieces of information available at whichever Sensing as a Service [20] platform, but they remain still not easily interoperable.

#### 2.1.2. Syntactic Interoperability Solutions

Syntactic level interoperability relates to the interoperation of the data structure and the format used for exchanging information between different IoT platforms. This kind of interoperability aims at smoothing message transition among different IoT systems. Syntactic interoperability can be achieved through pre-defined interfaces, data formats and encodings.

Middleware-like technologies have been typically applied in IoT research [21,22,23] to solve this problem. For example, [24] proposes a software gateway to dynamically map the physical devices in the home automation domain. Based on the mapping of the commands and parameters of the different platforms, one device from *platform A* can be discovered and controlled by a system on *platform B*. However, devices do not always know the proposed middleware specifications when they belong to different contexts. Thus, interoperability remains a problem.

The Collaborative Concept Exchange (CONEX) project devises a tree-alike XML syntax called XML Product Map (XPM) [25] to solve the cross-context syntactic interoperability problem. The key problem of this kind of interoperability solutions is that the uniformly described information still lacks from the necessary metadata to fully understand it and use it wisely. Following with the example in the previous section, these solutions will avoid the inconsistent format problems and partly the meaning one, but the service provider will still need to know if that information relates to the health, weather or car monitoring application domain.

#### 2.1.3. Semantic Interoperability Solutions

The use of semantic web technologies to query and manage information within federated cyber-infrastructures [26,27] is being explored as a promising approach to support the necessary coherence among heterogeneous experimental infrastructures. However, most of them make a top-down approach defining only the framework and assessing the meta-directory service using their own ontologies [28,29], or extensions of established ontologies such as the W3C Semantic Sensor Network (SSN) ontology [30]. Probably, the most widely used ontology is the Semantic Sensor Network (SSN) Ontology [31], which covers sensing, but does not take actuating or other realms of IoT into account. Moreover, this ontology is very complex to use at its full extend and is typically used as a baseline reference. The FIESTA-IoT ontology [32] uses the SSN as a basis and adds Architectural Reference Model (ARM) [15] key concepts to provide a more holistic IoT model. Other work that is pursuing parallel objectives and that is worth mentioning is oneM2M [33]. As an international partnership project of standardization bodies, it is defining a standard for M2M/IoT-communications. While the current release sets the base ontology to include semantic description of resources, it remains generic and the specification on how this can be applied and how interfaces will be extended to manage such information are still a work in progress and are being addressed as one major point for future releases.

The approach followed in this paper relies on this semantic interoperability approach but goes a step forward by taking into account the necessities from already deployed infrastructures, and defining the procedures for them to join their federations.

### 2.2. Applications of the Semantic Web enabled IoT

The Internet of Things (IoT) is unanimously identified as one of the main technology enablers for the development of future intelligent environments. It is driving the digital transformation of many different domains (e.g., mobility, environment, industry, healthcare, etc.) of our everyday life. One of the key drivers for this hype towards the IoT is its applicability to a plethora of different application domains [34], like e-health [35], smart cities [36], smart-home [37] or Industry 4.0 [38].

Employing semantics’ principles and methodologies to enhance IoT platforms address challenges of interoperability, data fusion, integration of heterogeneous IoT silos, annotation of data streams, just to name a few. In the area of e-health, data semantization is mostly used for providing healthcare services. For example, [39] proposes eHealth Recommendation Service System which recommends healthcare services for patients. In the area of smart homes, semantics have been used for data management and application [40]. Similarly, in [41] the knowledge reasoning features enabled by the use of semantics is exploited to support an smart factory environment. Finally, in the area of smart cities, this paper is actually a good example of the use cases that the use of semantics can enable.

### 2.3. Wise-IoT and BIG-IoT Platforms

As it has been mentioned in Section 1, the main technical novelty that is included in our work has been the integration of the system that enables interoperability between oneM2M, FIWARE and BIG-IoT platforms in order to support the use case that is described in this paper. The integrated system is using the solutions and components that have been developed in two European projects, Wise-IoT and BIG-IoT. This section summarizes the key features from these projects and briefs on the semantic domain models that are at the core of the integration of IoT platforms.

#### 2.3.1. Wise-IoT Key Features

The Worldwide Interoperability for Semantics IoT (Wise-IoT) project aimed at deepening the interoperability and interworking of existing IoT systems. Its main objective has been to propose a framework to facilitate the interoperability between various heterogeneous IoT platforms and protocols that were used in Europe and South Korea and offer portability through an information access layer interconnecting different platforms to achieve Global IoT Services (GIoTS). In particular, efforts during the project lifetime were devoted towards developing global standards to realize this global interoperability within the IoT ecosystem taking into account the complexity of the IoT standardization landscape. In order to do so, different use cases were implemented as a mechanism to extract requirements and to validate the outcomes. As a result, Wise-IoT allowed making significant progresses and increasing interoperability in different ecosystems, being the most interesting in the context of this paper the ones related to oneM2M and FIWARE based context information management.

**Figure 2:**
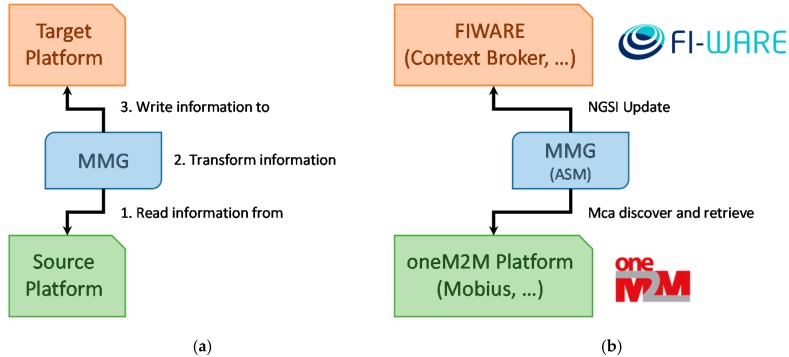
Abstract architecture of the MMG (**a**) and example of an MMG instantiation (**b**).

Clearly, it is unrealistic to fully homogenize the data and semantics model for each of the underlying platforms. The solution Wise-IoT adopted to overcome this problem has been to introduce an entity, known as Morphing Mediation Gateway (MMG), acting as a bridge between different domains. In essence, it is in charge of translating, at runtime, a representation of information from one domain to another. Those domains can either be different IoT platforms (e.g., oneM2M and FIWARE Orion Context Broker) or include IoT devices and communication technologies (e.g., LoRaWAN devices and oneM2M platform). As a result, MMG decouples the source platform / technology and the target platform using a modular architecture that allows the deployment of additional functionality on the fly. Figure 2 shows a simplified high-level overview of the behavior of this component (a) and its particularization for an oneM2M-FIWARE combination (b).

**Figure 3:**
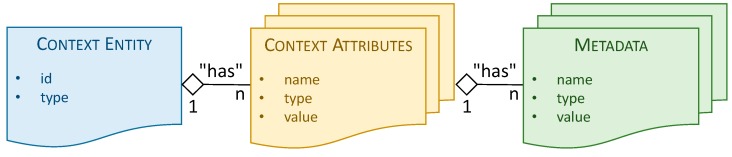
FIWARE NGSI context information model.

MMG component usage in the scope of this paper results in the generation of different FIWARE based context entities, which are structured following the schema depicted in Figure 3. *Context entities* are the key elements in the FIWARE NGSI information model, and represents a thing, which can be any physical or logical object. On the other hand, *context attributes* are the properties of a particular context entity, while *metadata* information can be used to describe properties of the attribute value. It is important to highlight that this structure is a generic abstraction of the different existing FIWARE data models [42]. Each of them instantiates the specific attributes and metadata fully defining the corresponding context entity. The equivalent information model used on an oneM2M system can be found in [43].

#### 2.3.2. BIG-IoT Key Features

The Bridging the Interoperability Gap of the IoT (BIG-IoT) project ambitions were to enable the emergence of cross-platform, cross-standard, and cross-domain IoT services and applications toward building IoT ecosystems. These ecosystems will connect thing providers, service providers, and users. To promote this IoT ecosystem, interoperability across platforms have to be supported. Developers will be able to create applications by combining data from multiple platforms even if these platforms come from multiple domains.

To enable interoperability for IoT platforms, the BIG-IoT API offers seven key functionalities. They cover resource registration and subsequent discovery of resources based on user-defined search criteria. Of course, access to data and metadata (data pull as well as publish-and-subscribe for data-streams) is supported. It also addresses vocabulary handling for semantic descriptions of resources. Finally, security management, which not only includes authentication and authorization, but also accounting and charging for enabling the monetization of assets. The other pillar of the BIG-IoT architecture is the BIG-IoT Marketplace. In this marketplace, IoT platforms and services providers can trade their resources (information and functions). IoT applications or services can use the marketplace to discover and access resources. The BIG-IoT Marketplace and API are the basis for providers and consumers to interact, exchange services, and monetize them.

The BIG IoT Semantic Application Domain Model purpose is to ensure semantic interoperability that bridges applications and services within BIG-IoT as well as across other Linked Data Platforms. The core concept in its domain model is the *Offering*. An *Offering* represents the resources or services that an IoT provider wants to export. The Application Domain Model comprises a core model, which defines the fundamental vocabulary required to create an Offering Description; and both domain independent and domain dependent models, which are used to further enrich the semantics of the Offering Descriptions.

**Figure 4:**
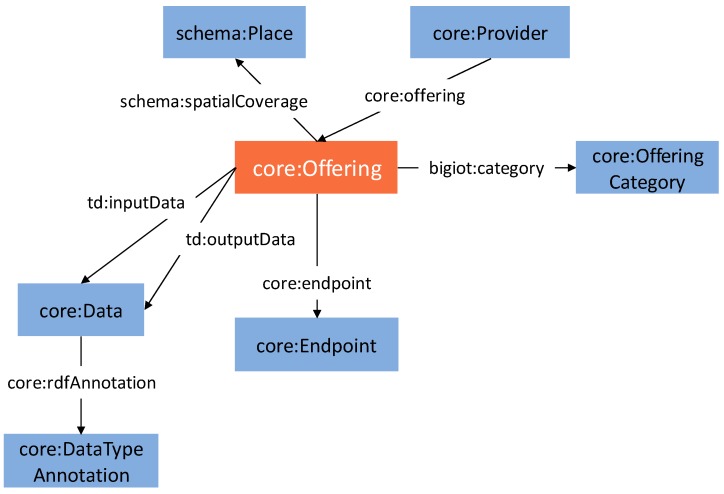
BIG-IoT Semantic Application Domain Model [44] excerpt.

The key concepts around the *Offering* one are presented in Figure 4. The input and output data and the offering category are key parts for the integration of BIG-IoT with other platforms. Firstly, because in the process of searching for offerings, the most important part of the query is probably the data being returned and required by an offering, but, principally, because these are the pieces of information that will have to be mapped from one domain model to another. Another important concept that is used for the discovery of offerings is the *OfferingCategory*. The BIG-IoT Marketplace is actually organized around it so it should be the first aspect to be used by IoT data consumers.

This generic model is made concrete through domain dependent vocabularies that BIG-IoT project has created following the style of the schema.org (https://schema.org/) project. The project has defined two vocabularies for Smart Mobility (http://schema.big-iot.org/mobility/) and Environmental Monitoring (http://schema.big-iot.org/environment/) domains respectively as they were the focus of the BIG IoT pilots.

## 3. IoT Infrastructure Involved in the Pilot

As it has been already introduced, fragmentation within IoT ecosystems is one of the key challenges to address for effectively deploying a GIoTS. In this sense, it seems obvious to think that different cities across the world will be deploying different IoT infrastructure to support and enhance the effectiveness of services provided to its citizens. This is also applicable within a single city, where different IoT infrastructures deployed at different moments have to coexist. This section provides an overview of the IoT devices and IoT platforms available within the cities of Santander (Spain); Busan, Seoul and Seongnam (South Korea); and Barcelona (Spain), which are specifically involved in the Smart Parking case described in this paper.

### 3.1. Santander Infrastructure

While most of the IoT infrastructure available in the city of Santander (Spain) has already been described in [45], new IoT deployments are occasionally added to the existing infrastructure. In this sense, additional infrastructure used for the use case described in this paper has been recently added. This section presents key details of the smart parking service infrastructure associated with this field trial.

**Figure 5:**
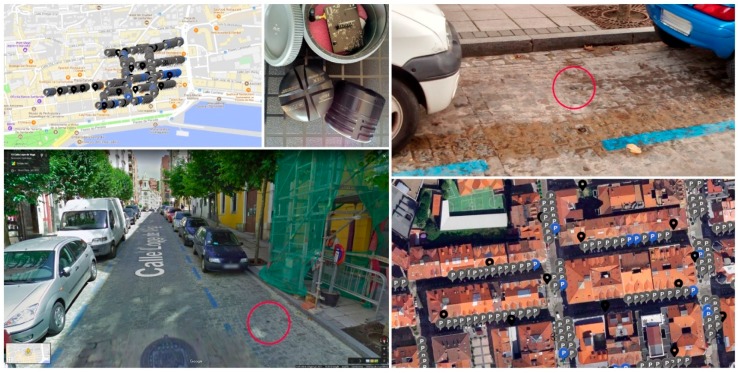
Santander Smart parking infrastructure in the city center area.

Being a smart parking trial, the pilot makes use of parking sensors already deployed in the city of Santander. In this regard, more than 250 outdoor parking sensors are installed in the main parking areas of the city center in order to detect parking site availability in these zones. Figure 5 includes a map of the area, which comprises more than 10 hectares, showing the installed parking infrastructure. These sensors, which are buried under the asphalt, are based on ferromagnetic detection and use the 868 Mhz band to transmit their status to the corresponding data collector. Figure 5 also shows some examples of installed devices. Data generated by these devices are transmitted to the manufacturer’s back-end and then, once processed, reinjected again in the Santander smart city platform in the form of free/occupied events per parking spot. As it will be presented later on in this section, different types of parking sensors are part of the pilot. To differentiate them, from now on these devices will be referred in this paper as legacy parking sensors.

**Figure 6:**
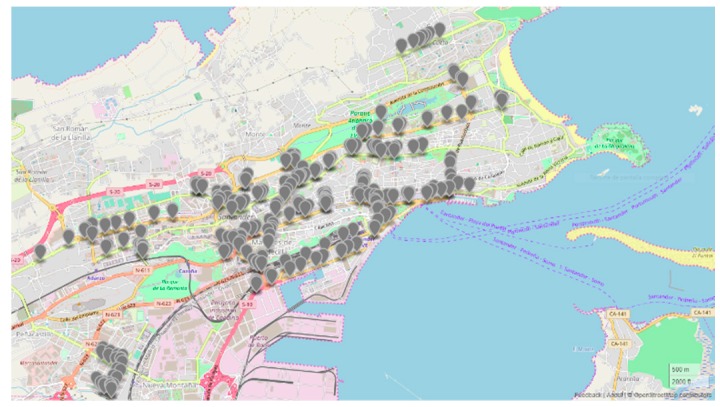
Map of magnetic loops location in the city of Santander.

Together with the aforementioned legacy parking sensors, the field trial also takes advantage of the different magnetic loops deployed across the city to model traffic congestion in the roads. This infrastructure is also used by the municipality for traffic management tasks and traffic lights control. The infrastructure is comprised of more than 300 IoT devices scattered throughout the whole city main roads (see Figure 6). The information provided by these sensors includes the percentage of time a vehicle is on top of the magnetic loop, the total counted vehicles per hour and a traffic congestion index which gives an estimation of the traffic status in that specific area.

As part of the work carried out for this field trial, both the legacy parking information and the traffic information are exported in real time from their specific IoT platforms (i.e., SmartSantander platform [46] and Santander Open Data platform (http://datos.santander.es)) into an instance of Orion Context Broker (https://fiware-orion.readthedocs.io). This component has been developed as part of the European Future Internet Platform FIWARE, according to Next Generation Service Interface (NGSI) standards.

**Figure 7:**
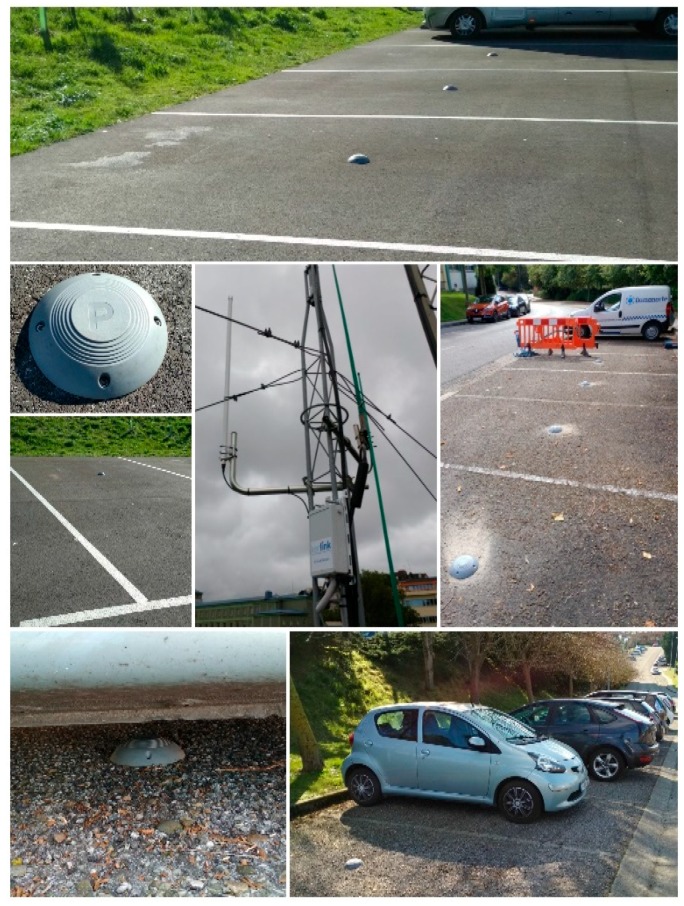
LoRa deployment examples in the city of Santander.

As it has previously been mentioned, this field trial aims at the combination of heterogeneous infrastructures to validate the concept of GIoTS in a fragmented scenario. Following this idea, we decided to accomplish the deployment of a new parking infrastructure based on Low-Power Wide-Area Network (LPWAN) communications, and more specifically on the LoRa (https://www.semtech.com/technology/lora) wireless technology. Even though smart parking sensors are provided by South Korean manufacturer SK Techx (Seoul, South Korea) as part of Busan smart city project, they are not the same ones as the ones deployed in the city of Busan, as we will see in the following subsection. A total of 35 sensors have been installed in an area close to the university campus. These sensors, which are mounted over the floor, are based on radar sensing and the communication range is around 1 km in urban areas. In order to support LoRaWAN (https://www.lora-alliance.org/technology) connectivity in the area, a Kerlink LoRa Wirnet Station (https://www.kerlink.com/product/wirnet-station) has been deployed on top of one of the university buildings and more gateways are expected to be installed on the city center in the months to come. Different pictures of the deployed LoRa based infrastructure can be seen on Figure 7.

For this pilot, the LoRa network included only one gateway. Therefore, and for the sake of simplicity, the handling of the whole LoRaWAN protocol stack is performed by a software layer installed on the own gateway instead of using a complete LoRaWAN infrastructure. This software, known as SPN (Small Private Network), enables a channel to send/receive information to/from the parking nodes. From that point on, different software components adapt and process the information to inject it into an instance of Mobius (http://developers.iotocean.org/archives/module/mobius), an open source IoT server platform based on the oneM2M standard.

In summary, from the entire IoT infrastructure currently available in the city of Santander, this field trial is based on parking and traffic sensor information. However, while legacy parking sensors and traffic sensors context information is available following a FIWARE based approach, LoRa parking sensors context information is available through oneM2M standard.

### 3.2. Busan, Seoul and Seongnam Infrastructure

Busan city, together with Goyang and Daegu, started using IoT technologies as part of the first generation of IoT-enabled smart city pilot project of the Ministry of Science and ICT (MSIT) in South Korea. In particular, during that pilot project (2015~2017), Haeundae-gu, one of the most developed districts in the city, carried out the deployment of the different IoT infrastructure and services focusing on general smart city services such as transportation and safety (http://www.k-smartcity.kr/english/index.php).

With the aim of enhancing interoperability, the use of a standard solution was an essential requirement since the planning phase. As a result, these three cities adopted the oneM2M IoT industry standard. OneM2M is an IoT middleware with standard interfaces for devices and applications for different IoT service domains, including smart city [47,48]. Fundamental features of smart city IoT platform like data exchange and security are well defined by oneM2M standard and it is also being used in commercial deployments. For data exchange, a rich set of APIs is supported for different service requirements. Historical data is natively supported within oneM2M platform. Security, including authentication and authorization, is another area in which oneM2M offers many different schemas that can be chosen by implementation systems.

During the last years, and due to lack of parking spaces that introduces major city problems like traffic jams, illegal parking and also contributes to an increment of the air pollution, most of the smart cities in different countries have deployed their own smart parking services. For the Busan pilot project, the parking service has been enhanced every year throughout its duration. In the first year, parking sensors were deployed on public parking lots to provide real-time parking service data. During the second year, CCTV based image recognition technology was implemented to get the occupancy data. Finally, during the last project’s year, parking spaces with electronic vehicle charging stations were also included.

**Figure 8:**
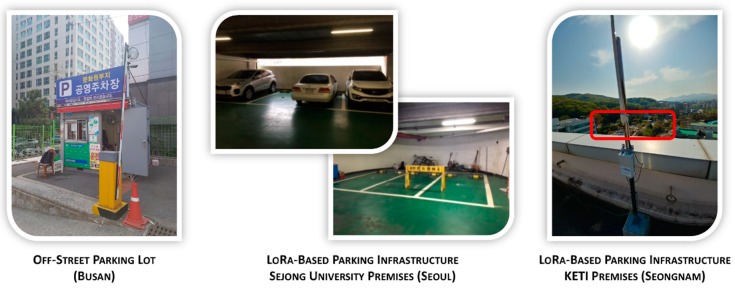
IoT parking deployment examples in the cities of Busan, Seoul and Seongnam.

Among the parking lots that provide parking data to the Busan smart city platform, six indoor ones have been chosen to be part of the smart parking use case within Wise-IoT project, for a total of 204 smart parking sensors. Figure 8 shows an example of one of those parking lots. They provide real-time occupancy data per parking spot using the global standard interfaces proposed by Wise-IoT framework for platform interoperability. As service roaming is one of the requirements of this use case, it is preferable to provide the same level of service data as Santander does. Hence, parking status information per spot is offered together with the total number of free spots in the parking lot, which is a more usual value for this situation.

Busan open platform can also be interworked with other oneM2M platforms, providing that both of them support the same oneM2M interfaces. This is the case of the Wise-IoT parking service deployment in Korea. Thus, in order to achieve oneM2M-FIWARE cross-domain interoperability, oneM2M standard semantic capabilities was required. However, as the Busan smart city platform do not provide them, another oneM2M compatible platform (Mobius) is used instead for the parking data.

On the other hand, and as part of its involvement in Wise-IoT project, Sejong University and KETI research institute carried out the deployment of a LoRaWAN based IoT parking infrastructure similar to the one deployed in Santander, which has been depicted on Section 3.1. A total of 36 parking sensors were deployed in Seoul and 35 parking sensors were deployed in Seongnam. Figure 8 shows different examples of these deployments.

### 3.3. Barcelona Infrastructure

**Figure 9:**
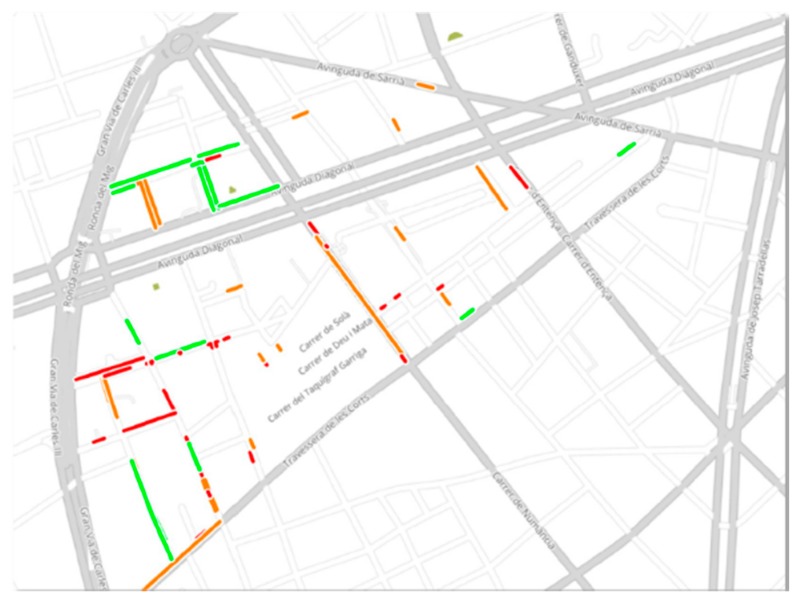
Parking detectors distribution in Barcelona.

Barcelona has achieved a wide range of benefits through investment in IoT for urban systems. Specifically, for the Smart Parking case, the city has invested in deploying a sensor system for drivers that guide them to open parking spots. Back in 2014, 600 wireless parking sensors from the company WorldSensing were deployed on the streets of Barcelona’s Les Corts district. The specific covered area can be seen in Figure 9. Embedded underneath the asphalt, the sensors can identify the available parking spaces and notify the drivers. The program is meant to reduce the emissions and congestion by directing drivers to vacant parking spaces.

The access to all the sensor data can be done through a proprietary API that the sensors’ vendor provides. Additionally, the developments carried out within the BIG-IoT project has made them available using the BIG IoT API.

## 4. Use-Case Requirements

The smart parking use case that we are presenting in this paper aims at demonstrating how it is possible to consume data from five different and heterogeneous smart city deployments (presented in the previous section). The main objective of the field trial presented in this paper is to serve as a validator of GIoTS, enabling transparent user roaming between the five cities involved in the pilot.

The system that has been integrated in order to support this use case and its main objective had to address a number of requirements. These requirements defines the main design considerations that we have observed during the development of the underlying integrated system. Moreover, these requirements have also conditioned the components that we have used for the integrated system.

The key requirements that have been fulfilled as well as how they are bound to the key features and components used from Wise-IoT and BIG-IoT platforms are as follows:

*Interoperability*: Often referred as interworking, it represents the ability of systems to connect, communicate and exchange information. In a domain where several vendors are expected to coexist, access to context information from heterogeneous platforms is necessary in order to overcome vendor lock-in situations. Interoperability implies adherence to standards, translation between systems built with different ones, and establishing agreements to share data via well-known interfaces in order to facilitate data sharing and exploitation. In this sense, different options can be considered, being two of the most interesting ones:

- to replicate IoT data on the different IoT platforms, leveraging the existing knowledge around the existing ones. In principle, the response time would not be heavily impacted by the addition of new heterogeneous nodes. However, this solution demands higher capacity, which could lead to scalability problems in the future if the data set grows in an unbounded way.
- to discover and translate the IoT data in real time, always using home IoT platforms to retrieve the information and avoiding duplication, hence adapting not only the underlying IoT data but also the operations allowed by the different interfaces. Nevertheless, this would result in extra complexity and a considerable increase in the overall system response time.

*IoT data abstraction and integration*: Data abstraction in IoT is related to the manner in which the physical world data is represented and handled. It is required to have a set of constructs to formally describe not only the sensor resources but also the sensor observation and measurement data. With the semantic descriptions, the IoT data can be characterized on different abstraction levels or merged with other information to generate different views of the environment. Moreover, it can also be incorporated to the data analytics mechanisms to leverage context awareness. However, mapping among the used semantic models is still necessary to support the integration of cross-platform IoT data that might be represented using different domain knowledge models.

*Services Exportability*: The challenges that cities face are quite common to any of them. Thus, the smart services that improve the efficiency of the city and the well-being its citizens are potentially beneficial to any city on which they are provided. This is the main motivation for service providers and application developers to invest resources in developing such services. However, if the process of replicating the service deployment is not seamless among different cities, the profit margins are jeopardized by the expenses involved in tailoring the solution to the specific infrastructure and platform that is deployed in each city. Only if the same service and or application can be seamlessly used across cities, thus creating GIoTS, the full potential of IoT-based smart cities will be reached.

*Discoverability*: It is not enough with setting the mechanisms to make data interoperable among IoT Platforms, but it is also necessary to define the ecosystem where the different platforms can advertise their datasets and services. Additionally, this ecosystem should also be able to track the interactions between providers and consumers of data and/or services. This tracking should consist on authentication and authorization (i.e., to guarantee that actors in the ecosystem are authorized to actuate) but also accounting and charging (i.e., to support monetization of transaction of services). This ecosystem should resemble a marketplace where application developers can discover available context information and then they can be charged by the providers that have advertised and offered their services.

*Programmability*: Creating the ecosystem is the necessary but not sufficient condition. A fundamental risk of such ecosystem is that developers and platform providers might find the ecosystem’s features unattractive. For the ecosystem to grow, developers must find it easy to interact with the interfaces offered. Provider and consumer should be equipped with software libraries that eases the process of interacting with the ecosystem functionalities. For example, the provider library implements a registry interface to offer resources through the marketplace and access interfaces to provide the information to consumers. These libraries support platform, service, and application developers in trading, discovering, and accessing resources.

## 5. IoT Platforms Interoperability

As it has been described in Section 3, parking sensor observations generated in Santander are distributed on different instances of oneM2M and FIWARE-based platforms, while information generated in Busan, Seoul and Seongnam uses oneM2M as back-end and Barcelona’s infrastructure can be accessed using the interfaces defined by the BIG-IoT project. Therefore, cross-domain interoperability between oneM2M, FIWARE and BIG-IoT platforms has been necessary in order to enable the implementation of the smart parking trial. This section summarizes the system that has been integrated leveraging platform interoperability solutions from the Wise-IoT and BIG-IoT projects to tackle this problem. This system provides the necessary back-end to support the implementation of the pilot described in this paper.

**Figure 10:**
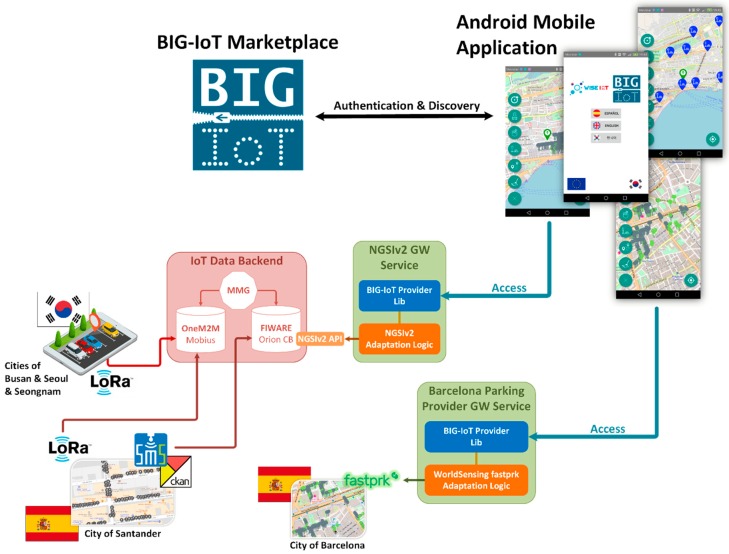
Platform interoperability high-level architecture.

Firstly, Wise-IoT project aims to create a comprehensive mediation framework that can be used between FIWARE and oneM2M IoT platforms. This is achieved through the development of a semantic model to enable interoperability at the data level, thus reducing the effort needed to develop new applications and services. Information available on each platform is automatically discovered, translated and injected into the other one. Consequently, services only need to interact with a single IoT platform and same-domain data following different standards can reuse the same information models. The conceptual layer that abstracts the underlying IoT platforms to provide a single one based on multiple standards is known as Information Access Layer. The entity in charge of this interconnectivity task is the Morphing Mediation Gateway (MMG) [49], and more specifically the Adaptive Semantic Module (ASM). ASM is responsible of the translation from an oneM2M source platform to a FIWARE-based one using the available oneM2M semantic annotations as depicted in Figure 10. Additionally, data from Santander, Busan, Seoul and Seongnam cities related to parking are built following the correspondent Wise-IoT data model [50] and translated by the Wise-IoT Morphing Mediation Gateway to be finally stored on the Information Access Layer.

The MMG enables seamless access to the information available from the Santander’s, Busan’s, Seoul’s and Seongnam’s deployments, however it was still necessary to get information from the Barcelona’s sensors. In order to finally homogenize the access to the five infrastructures, a second conversion was necessary. This second conversion relied on the architecture and platform provided by the BIG-IoT project [4].

According to the categorization made considering the implementation of the BIG-IoT API and the integration with marketplaces, the FIWARE-based platform that enables the access to Santander’s and South Korea cities’ sensors can be modelled as a Type 1 platform [51]. In this sense, it can be considered as being “always online” and having sufficient compute and memory resources to implement and offer Web based APIs for the interaction with the marketplace and other applications or services (as consumers).

Further to this categorization, which mainly establishes the availability of the offerings to be published at the BIG-IoT Marketplace by the underlying IoT platform, the most relevant design decision for the integration of the FIWARE-based platform is the Integration Mode (IM) [51] to be used according to the options defined by the BIG-IoT architecture. Taking into account that the interfaces of the FIWARE-based platform are already standard, the option chosen is the IM 2. For this IM a Gateway Service has been developed and operated. It handles all authentication and discovery interactions made through the BIG-IoT Marketplace and translates the access requests into the corresponding NGSIv2 calls to retrieve the demanded context information. The *Query* operation from the FIWARE-NGSIv2 API [52] has been employed. In this sense, the offerings published in the BIG-IoT Marketplace will work in ACCESS mode [53].

**Figure 11:**
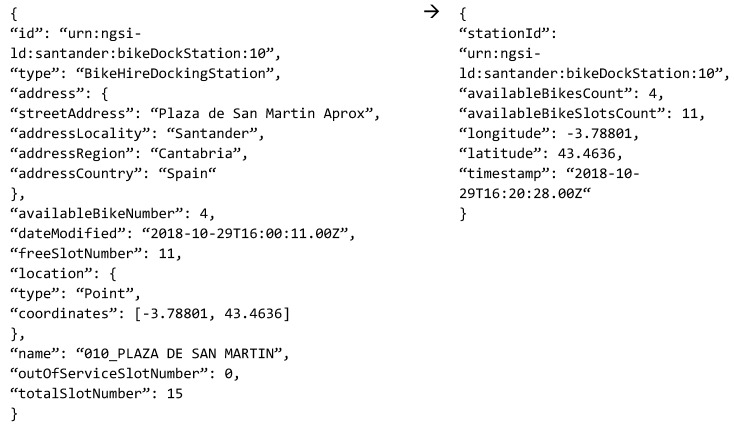
Data models transformation example.

Internally, the GW Service makes the transformation between the two data models (i.e., FIWARE and BIG-IoT ones). Figure 11 shows an example of the transformation that happens internally at the Adaptation Logic. Additionally, the Java classes’ development has been done in such a way that tailoring the GW Service to the specific features of any FIWARE-based IoT deployment can be done in a straightforward manner.

It is important to highlight that, as it has been already introduced in Section 2, while FIWARE has fixed data models for each domain, the BIG-IoT platform does not impose any syntactic restriction to the input and output data of its offerings. Focus of the BIG-IoT Semantic Core Model is put on the discoverability and accessibility of the offerings. Thus, the Offering Description objects’ model is generically defined to fit on any application domain. Input and output data objects from a BIG-IoT offering are not bound to use pre-defined attributes. Context providers can declare their offerings as they best prefer. For addressing interoperability in specific domains (such as mobility, environment, traffic, parking, etc.), the model defines an *rdfAnnotation* attribute which is used to reference the appropriate type within the BIG-IoT schema.org taxonomy. The mapping between the two data models have been done by establishing *equivalentTo* object properties for the attributes defined in the FIWARE data models to bind to the specific *rdfAnnotation* of the BIG-IoT schema.org taxonomy’s categories. This way, when the consumers use the BIG-IoT Marketplace for discovering the context information accessible through the existing offerings, they can use the semantics introduced in the *rdfAnnotation* included on each input and output data objects to look for the pieces of context data in which they are interested.

The GW Service that we have implemented manages internally the conversion so that consumers can homogeneously discover and access the information whether it has been originated in an oneM2M platform, a FIWARE-based one or directly using the BIG-IoT API. As it can be seen in Figure 10, for the deployments that are directly integrated into the BIG-IoT Marketplace (e.g., Barcelona’s one) only the proprietary adaptation logic is necessary to consume the IoT data using the BIG-IoT API. Those infrastructures, like SmartSantander one, relying on the Orion Context Broker (https://fiware-orion.readthedocs.io/en/master/) (the implementation of the NGSIv2 REST API binding that we have employed in the system that we have integrated) are discoverable and accessible through the GW Service that we have implemented. Finally, the deployments from South Korea, and a part of the SmartSantader infrastructure, that exposes their services through Mobius (the oneM2M reference implementation used in the system that we have integrated), are also discoverable and accessible through the GW Service that we have implemented. However, in this last case, the MMG has previously replicated the actual oneM2M services within the Orion Context Broker.

Once the GW Service for the FIWARE-based platform has been implemented, all the offerings from Santander, Busan, Seoul, Seongnam and Barcelona were registered at the BIG-IoT Marketplace (https://market.big-iot.org/) so that they can be discoverable and accessible for authenticated applications. The mobile App that was implemented for supporting the parking use case, acted as one of these applications consuming the information provided by these offerings. After getting the authentication token, discover the relevant offerings’ endpoints, and the input and output parameters for each of them, the App is able to seamlessly consume the data from any of them obtaining the results in a both syntactic and semantic uniform manner. Thus, transparently getting context information from the five cities.

## 6. End-User Application

From a user perspective, the underlying heterogeneity is actually hidden. They interact with the field trial through the application that has been implemented. In this sense, the smart parking use case is based on a mobile App that has been implemented to enhance the mobility experience in the city.

Before delving any deeper into the description of this application, it is important to mention that already existing parking applications actually provide features that can cover basic needs such as presenting the available parking lots. However, none of them currently provides additional characteristics to make the parking experience lighter (e.g., providing routes with less traffic to arrive). Additionally, these applications are usually limited to specific areas, having to change the application when you move to another city.

The objective of this use case is to provide users with services and applications that can be used in Seongnam, Seoul, Busan, Barcelona and Santander (actually from any city providing parking information at the BIG-IoT Marketplace), exploiting the interoperability features provided by the semantic-enabled system described in Section 2. As a result, same services can run over the application transparently using data from different sources.

As an outcome of this use case one Android application has been developed as part of our work for Wise-IoT and BIG-IoT projects. This application discovers the available information (related to on-street parking availability, traffic conditions and public bicycles stations status) from the BIG-IoT Marketplace and then gets it through the GW Service that was implemented to wrap the FIWARE Orion Context Broker that manages all the information in the backend. The mobile application, known as BigCitiesSmartMobility App, is focused on providing useful functionalities to car drivers at two different stages: (a) when drivers are seeking for a free parking spot near a selected area, and (b) when drivers have already parked their car.

**Figure 12:**
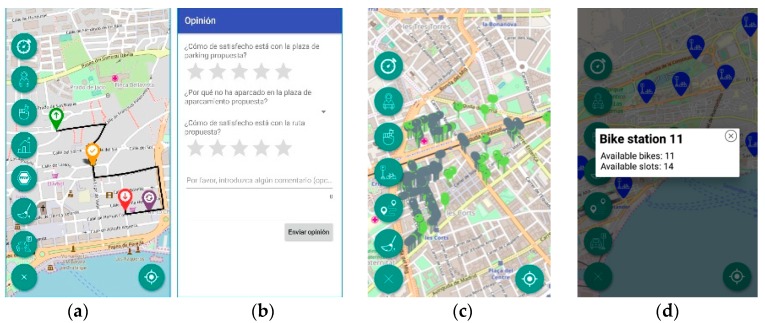
BigCitiesSmartMobility App mobile application features. (**a**) guidance to nearest available parking place; (**b**) experience feedback collection; (**c**) available and occupied parking spaces; (**d**) location of bike stations and availability of public bikes.

The main feature offered during the first phase is route provisioning. In this context, routes based on both on-street and off-street parking are considered. It is important to mention that after the semantic interoperability techniques have been applied to the information generated by the underlying sensors, both kind of parking places can be seamlessly discovered. Additionally, meta-data about the parking spot is also uniformly provided so that the application can consume it and adapt the guidance to the user preferences (e.g., cost, indoor or outdoor, etc.). In order to obtain a route, the users have to select a starting point (based on their current GPS position or manually) and, optionally, a desired destination area (users’ surroundings are used if target area is not explicitly specified). Taking this inputs into account the application calculates a route (Figure 12a) observing other parameters such as traffic congestion, parking occupancy, distance, etc. This is possible due to the different Wise-IoT recommendation services [54], which base their calculations on the data generated by the IoT infrastructure described in Section 3. Once monitoring is stopped (when drivers reach their destination), users are invited to provide feedback, and the requested information differs based on the results derived from the monitoring (see Figure 12b). Besides route calculation, users can also have access to real time status and statistical information of the monitored parking spots as it can be seen in Figure 12c, which also illustrates the roaming scenario when the application is used in Barcelona.

Once the drivers have parked their cars, the application completes the smart mobility case by providing (a) information about public bicycles stations status; (b) walking routes back to the car (if the position has been previously stored), and (c) manual reminder of parking ticket expiration via mobile notifications. An example of (a) is provided in Figure 12d.

As mentioned, the features from the application can be provided in a roaming scenario (i.e., driver travelling from Santander to Barcelona, for example). This is possible due to the already introduced data interoperability, as it allows gathering, managing and presenting the information that the infrastructure deployed in the cities generate in the application.

## 7. Concluding Remarks

The IoT paradigm, being one of the technologies that are meant to transform our future way of living, brings about an important issue that is still not properly solved: how to guarantee interoperability among the existing (and still growing) IoT platforms. The fragmented IoT landscape that the lack of such an interoperable IoT creates is behind two major concerns that are preventing, particularly in the Smart City application scenario, further investments in IoT. These two fears are the vendor lock-in and the city lock-in. Besides the actual process of technology maturation, these concerns are somehow hampering the IoT market uptake both for infrastructure vendors and for smart service providers.

Among the initiatives that are being explored to tackle the IoT interoperability challenge there are two IoT platforms, FIWARE and oneM2M that are already considering the application of semantics to their interfaces and information models so that semantic mediation gateways can be used to make them interoperate through the use of semantics. Additionally, both approaches are creating standards on ETSI (https://www.etsi.org/about/what-we-do/global-collaboration/onem2m) (https://www.etsi.org/deliver/etsi_gs/CIM/001_099/004/01.01.01_60/gs_CIM004v010101p.pdf). However, even when standardization efforts are being carried out, it is not foreseen that the future IoT scenarios will be supported by only one standard. The fact that already some of the pioneering solutions (e.g., FIWARE one) have a significant critical mass in terms of the number of followers and community that they have, augurs an IoT landscape that will remain heterogeneous.

This paper has presented a global smart parking use case carried out in five different cities around the world: Seongnam, Seoul, Busan, Barcelona and Santander. For supporting this field trial, we have integrated three different platforms leveraging the semantic interoperability components provided by two research projects, namely Wise-IoT and BIG-IoT. The MMG from Wise-IoT enabled homogeneous access to the sensor readings coming from the IoT deployments at Santander, Busan, Seoul and Seongnam, which respectively used FIWARE-NGSI and oneM2M platforms. However, the FIWARE-NGSI platform only provides the API for accessing to the available context information. To make the solution more user-friendly for application developers, it is necessary to set the marketplace where all this data can be discovered. The BIG-IoT marketplace (https://market.big-iot.org/) has been used to fulfil this requirement. An NGSIv2 GW Service has been implemented to support the publication of context data, available through FIWARE-based platforms, as BIG-IoT marketplace offerings.

By chaining the MMG and the NGSIv2 GW Service, the BIG-IoT marketplace could be populated by offerings exposing data from Santander (FIWARE-based platform with some infrastructure using oneM2M-based platform), Busan, Seoul and Seongnam (oneM2M-based platform). Additionally, other parking related information was already available at the marketplace so the use case could benefit from it. In the use case that has been described in this paper, the data from the parking sensors deployed in one of the districts of the city of Barcelona (proprietary platform) was also employed.

The main contribution from this paper has been the integration of the abovementioned three platforms using semantic interoperability capabilities from Wise-IoT and BIG-IoT solutions in order to support the provision of global IoT services. At the same time, we have validated some of the components and concepts provided by the framework that these projects have proposed. Finally, thanks to this cross-city use case deployment, a smart mobility application can be used on the five different locations provided that on each of them the deployed infrastructure is based on a different underlying IoT platform.

## References

[1] Veer H., Wiles A. Achieving Technical Interoperability-the ETSI Approach. http://www.etsi.org/images/files/ETSIWhitePapers/IOPwhitepaperEdition3final.pdf.

[2] Uschold M., Menzel C. Semantic Integration & Interoperability Using RDF and OWL. https://www.w3.org/2001/sw/BestPractices/OEP/SemInt/.

[3] Sotres P., Lopez de la Torre C., Sanchez L., Jeong S., Kim J. Smart City Services Over a Global Interoperable Internet-of-Things System:The Smart Parking Case. Proceedings of the 2018 Global Internet of Things Summit (GIoTS).

[4] Broring A., Schmid S., Schindhelm C.-K., Khelil A., Kabisch S., Kramer D., Le Phuoc D., Mitic J., Anicic D., Teniente E. (2017). Enabling IoT Ecosystems through Platform Interoperability. IEEE Softw..

[5] Aloi G., Caliciuri G., Fortino G., Gravina R., Pace P., Russo W., Savaglio C. (2017). Enabling IoT interoperability through opportunistic smartphone-based mobile gateways. J. Netw. Comput. Appl..

[6] Ide N., Pustejovsky J. What does interoperability mean, anyway? Toward an operational definition of interoperability for language technology. Proceedings of the Second International Conference on Global Interoperability for Language Resources.

[7] Wang W., Tolk A., Wang W. The levels of conceptual interoperability model: Applying systems engineering principles to M&S. Proceedings of the 2009 Spring Simulation Multiconference.

[8] Shelby Z., Bormann C. (2011). 6LoWPAN: The Wireless Embedded INTERNET.

[9] Dujovne D., Watteyne T., Vilajosana X., Thubert P. (2014). 6TiSCH: Deterministic IP-enabled industrial internet (of things). IEEE Commun. Mag..

[10] IPv6 over Networks of Resource-Constrained Nodes (6lo). https://datatracker.ietf.org/wg/6lo/about/.

[11] RFC 6550 RPL: IPv6 Routing Protocol for Low-Power and Lossy Networks. https://tools.ietf.org/html/rfc6550.

[12] Aijaz A., Su H., Aghvami A.-H. (2015). CORPL: A Routing Protocol for Cognitive Radio Enabled AMI Networks. IEEE Trans. Smart Grid.

[13] Bellavista P., Giannelli C., Lanzone S., Riberto G., Stefanelli C., Tortonesi M., Bellavista P., Giannelli C., Lanzone S., Riberto G. (2017). A Middleware Solution for Wireless IoT Applications in Sparse Smart Cities. Sensors.

[14] Liu Q., Ma Y., Alhussein M., Zhang Y., Peng L. (2016). Green data center with IoT sensing and cloud-assisted smart temperature control system. Comput. Netw..

[15] Almeida R., Oliveira R., Luís M., Senna C., Sargento S., Almeida R., Oliveira R., Luís M., Senna C., Sargento S. (2018). A Multi-Technology Communication Platform for Urban Mobile Sensing. Sensors.

[16] Suárez-Albela M., Fernández-Caramés T., Fraga-Lamas P., Castedo L., Suárez-Albela M., Fernández-Caramés T.M., Fraga-Lamas P., Castedo L. (2017). A Practical Evaluation of a High-Security Energy-Efficient Gateway for IoT Fog Computing Applications. Sensors.

[17] Bhatkal A.P., Bharati K. (2014). A survey on improved framework for smart phone using internet of things. Int. J. Sci. Res..

[18] Aloi G., Caliciuri G., Fortino G., Pace P. A smartphone-centric approach for integrating heterogeneous sensor networks. Proceedings of the 9th International Conference on Body Area Networks.

[19] Gaggioli A., Pioggia G., Tartarisco G., Baldus G., Corda D., Cipresso P., Riva G. (2013). A mobile data collection platform for mental health research. Pers. Ubiquitous Comput..

[20] Perera C., Zaslavsky A., Christen P., Georgakopoulos D. (2014). Sensing as a service model for smart cities supported by Internet of Things. Trans. Emerg. Telecommun. Technol..

[21] Gama K., Touseau L., Donsez D. (2012). Combining heterogeneous service technologies for building an Internet of Things middleware. Comput. Commun..

[22] He W., Xu L. (2014). Da Integration of Distributed Enterprise Applications: A Survey. IEEE Trans. Ind. Inform..

[23] Kim D., Lee C., Park J.H., Moon K., Lim K. (2007). Scalable message translation mechanism for the environment of heterogeneous middleware. IEEE Trans. Consum. Electron..

[24] Moon K.-D., Lee Y.-H., Lee C.-E., Son Y.-S. (2005). Design of a universal middleware bridge for device interoperability in heterogeneous home network middleware. IEEE Trans. Consum. Electron..

[25] Guo J. (2009). Collaborative conceptualisation: Towards a conceptual foundation of interoperable electronic product catalogue system design. Enterp. Inf. Syst..

[26] Willner A., Giatili M., Grosso P., Papagianni C., Morsey M., Baldin I. (2017). Using Semantic Web Technologies to Query and Manage Information within Federated Cyber-Infrastructures. Data.

[27] Avgeris M., Kalatzis N., Dechouniotis D., Roussaki I., Papavassiliou S. Semantic Resource Management of Federated IoT Testbeds. Proceedings of the 16th International Conference on Ad-Hoc Networks and Wireless.

[28] Tachmazidis I., Batsakis S., Davies J., Duke A., Vallati M., Antoniou G., Clarke S.S. A Hypercat-enabled semantic Internet of Things data hub. Proceedings of the 16th European Semantic Web Conference.

[29] D ’elia A., Viola F., Azzoni P. (2017). Enabling Interoperability in the Internet of Things: A OSGi Semantic Information Broker Implementation. Int. J. Semant. Web Inf. Syst..

[30] Petrolo R., Loscrì V., Mitton N. (2017). Towards a smart city based on cloud of things, a survey on the smart city vision and paradigms. Trans. Emerg. Telecommun. Technol..

[31] Semantic Sensor Network Ontology. https://www.w3.org/TR/vocab-ssn/.

[32] Agarwal R., Fernandez D.G., Elsaleh T., Gyrard A., Lanza J., Sanchez L., Georgantas N., Issarny V. Unified IoT ontology to enable interoperability and federation of testbeds. Proceedings of the IEEE 3rd World Forum on Internet of Things (WF-IoT).

[33] Swetina J., Lu G., Jacobs P., Ennesser F., Song J. (2014). Toward a standardized common M2M service layer platform: Introduction to oneM2M. IEEE Wirel. Commun..

[34] Al-Fuqaha A., Guizani M., Mohammadi M., Aledhari M., Ayyash M. (2015). Internet of Things: A Survey on Enabling Technologies, Protocols, and Applications. IEEE Commun. Surv. Tutor..

[35] Islam S.R., Kwak D., Kabir M.H., Hossain M., Kwak K.S. (2015). The Internet of Things for Health Care: A Comprehensive Survey. IEEE Access.

[36] Afzal M.K., Rehmani M.H., Pescape A., Kim S.W., Ejaz W. (2017). IEEE Access Special Section Editorial: The New Era of Smart Cities: Sensors, Communication Technologies, and Applications. IEEE Access.

[37] Risteska Stojkoska B.L., Trivodaliev K.V. (2017). A review of Internet of Things for smart home: Challenges and solutions. J. Clean. Prod..

[38] Chen B., Wan J., Shu L., Li P., Mukherjee M., Yin B. (2018). Smart Factory of Industry 4.0: Key Technologies, Application Case, and Challenges. IEEE Access.

[39] Lee H.J., Kim H.S. eHealth Recommendation service system using ontology and case-based reasoning. Proceedings of the 2015 IEEE International Conference on Smart City/SocialCom/SustainCom (SmartCity).

[40] Tao M., Ota K., Dong M. (2017). Ontology-based data semantic management and application in IoT- and cloud-enabled smart homes. Future Gener. Comput. Syst..

[41] Wang S., Wan J., Li D., Liu C., Wang S., Wan J., Li D., Liu C. (2018). Knowledge Reasoning with Semantic Data for Real-Time Data Processing in Smart Factory. Sensors.

[42] FIWARE Foundation FIWARE Data Models. https://www.fiware.org/developers/data-models/.

[43] Gilani K., Kim J., Song J., Seed D., Wang C. (2018). Semantic Enablement in IoT Service Layers—Standard Progress and Challenges. IEEE Internet Comput..

[44] BIG-IoT Project Deliverable D4.2.b—Semantic Model for the Application Domain. http://big-iot.eu/wp-content/uploads/2016/04/D4.2.b_Semantic_Interoperability_Design_for_Smart_Object_Platforms_and_Services_Final_.pdf.

[45] Lanza J., Sánchez L., Gutiérrez V., Galache J., Santana J., Sotres P., Muñoz L. (2016). Smart City Services over a Future Internet Platform Based on Internet of Things and Cloud: The Smart Parking Case. Energies.

[46] Sotres P., Santana J.R., Sanchez L., Lanza J., Munoz L. (2017). Practical Lessons From the Deployment and Management of a Smart City Internet-of-Things Infrastructure: The SmartSantander Testbed Case. IEEE Access.

[47] Smart Cities Done Smarter, OneM2M Whitepaper 2017. http://www.onem2m.org/images/files/oneM2M_WhitePaper_SmartCitiesDoneSmarter.pdf.

[48] NIST IoT Enabled Smart City (IES-City) Framework. https://pages.nist.gov/smartcitiesarchitecture.

[49] Wise-IoT Project Deliverable 2.2: Morphing Mediation Gateway with Management and Configuration Functions R2. http://wise-iot.eu/wp-content/uploads/2018/03/D2.2-Morphing-Mediation-Gateway-with-Management-and-Configuration-Functions-R2-v1.0.pdf.

[50] Wise-IoT Project Deliverable 2.5: Semantic Interoperability Components R2. http://wise-iot.eu/wp-content/uploads/2017/11/D2.5-Semantic-Interoperability-Components-R2-v1.0.pdf.

[51] Schmid S., Bröring A., Kramer D., Käbisch S., Zappa A., Lorenz M., Wang Y., Rausch A., Gioppo L. An architecture for interoperable IoT Ecosystems. Proceedings of the International Workshop on Interoperability and Open-Source Solutions.

[52] Cantera-Fonseca J.M., Galán-Márquez F., Jacobs T. FIWARE-NGSI v2 Specification. https://orioncontextbroker.docs.apiary.io/#.

[53] Bröring A., Ziller A., Charpenay V., Schmid S., Thuluva A., Anicic D., Zappa A., Linares M.P., Mikkelsen L.M., Seidel C. (2018). The BIG IoT API—Semantically Enabling IoT Interoperability. IEEE Pervasive Comput..

[54] Wise-IoT project Deliverable 2.6: Self-Adaptive Recommendation System. http://wise-iot.eu/wp-content/uploads/2017/08/D2.6-Self-Adaptive-Recommendation-System-V1.02.pdf.

